# Association Between Expressed Emotion and Relapse of Bipolar Disorder: A Systematic Review

**DOI:** 10.31083/AP47961

**Published:** 2026-06-02

**Authors:** Sum Yu Tong, Moses Chung Hei Dye, Anthony Ho Tsun Yue, Yan Li, Dennis Chak Fai Ma

**Affiliations:** ^1^School of Nursing, Faculty of Health and Social Sciences, The Hong Kong Polytechnic University, Hung Hom, Hong Kong SAR, China; ^2^Department of Rehabilitation Sciences, Faculty of Health and Social Sciences, The Hong Kong Polytechnic University, Hung Hom, Hong Kong SAR, China

**Keywords:** bipolar disorder, family, expressed emotion, recurrence

## Abstract

**Background:**

Expressed emotion (EE) is a robust predictor of relapse in individuals with schizophrenia-spectrum disorders. However, the evidence on this relationship has not been fully examined in individuals with bipolar disorder (BD). This systematic review was conducted to provide updated evidence on the association between EE and relapse in BD.

**Methods:**

A systematic search was conducted in seven databases and two registries from inception to 29 December 2025. The Joanna Briggs Institute's (JBI) critical appraisal checklists were used to assess the risk of bias in the included studies. The review process was conducted independently by two reviewers, with consensus for each step achieved through consultation with a third reviewer. A narrative synthesis was performed to categorise the findings, with particular focus on the impact of global EE and its subdomains on relapse in BD.

**Results:**

Of the 2963 records identified, seven studies were included in this review. The majority of studies were rated as having a low risk of bias. The narrative synthesis indicates that high EE is significantly associated with an increased risk of relapse in BD, particularly for depressive episodes, and is linked to critical comments and emotional over-involvement, two key subdomains of EE.

**Conclusions:**

This review provides evidence of a potential association between high EE, its subdomains, and relapse in BD. These preliminary findings contribute to the development of family-based interventions for BD and highlight the need for larger-scale, longitudinal prospective cohort studies to further clarify this relationship.

**Registration:**

The study has been registered on https://www.crd.york.ac.uk/prospero/ (registration number: CRD42025632303; registration link: https://www.crd.york.ac.uk/PROSPERO/view/CRD42025632303).

## Main Points

• High expressed emotion, particularly critical comments and emotional over-involvement, is potentially associated with an increased risk of relapse in bipolar disorder.

• High expressed emotion is more likely to be linked with depressive episodes than with manic relapses in bipolar disorder.

• Development of family-focused therapies to modify components of expressed emotion may lower relapse rates and improve quality of life for individuals with bipolar disorder.

• More primary studies are required to clarify the impact of specific domains in expressed emotions and their mechanisms on the relapse of bipolar disorder.

## 1. Introduction

Bipolar disorder (BD) is a condition that is estimated to have affected over 0.5% of the world population in 2019 [1]. Individuals with BD experience periods of mood disturbance, which may be accompanied by depressive episodes and are associated with severe psychosocial and occupational dysfunction [2, 3]. Intensive treatments such as augmentation of antipsychotics and benzodiazepines with mood stabilizers, are used to treat the acute or refractory condition [4]. However, despite the treatment options, more than 90% of individuals with BD experience relapse in their lifetime, especially within two years of the initial occurrence of symptoms [5]. Relapse in individuals with BD further worsens their condition and diminishes their quality of life. A study conducted by Konno *et al*. [6] revealed that individuals experiencing manic and depressive episodes had a higher likelihood of unemployment. Additionally, the recurrence of symptoms in individuals with BD has been linked to impaired family relationships, frequent hospitalization and increased suicidal risk [7, 8]. Given the negative consequences brought by relapse, identifying modifiable risk factors that trigger relapses of BD allows for the development of new interventions to prevent relapses in the long term. Several modifiable risk factors related to sociodemographic and clinical aspects have been found to influence the relapse rate of BD. For instance, treatment non-adherence has been reported as a significant risk factor for predicting relapses in many studies [9, 10, 11]. Other factors such as disruption of circadian rhythm, unemployment, low education level and alcohol misuse are also found to be associated with an increased chance of relapse in BD [12, 13, 14, 15].

In addition to sociodemographic and clinical predictors, family-related factors have emerged as critical and modifiable influences on relapse in BD. Among these, expressed emotion (EE) is one of the family-related factors that has been extensively emphasised in recent decades for its impact on relapse. EE is described as the attitude of relatives or close friends towards an individual with a psychiatric disorder, which involves critical comments, hostility, positive remarks, warmth and emotional over-involvement [16, 17]. To assess the level of EE in family members, several assessment instruments, such as the Camberwell Family Interview (CFI), Five Minutes Speech Sample and Expressed Emotion Scale, are available. Among these, the Camberwell Family Interview is considered to be the most reliable assessment tool [18, 19]. Using a semi-structured interview format, the Camberwell Family Interview aims to assess the emotional climate between a key relative and patient with a mental disorder and hence classify the relative into high EE or low EE [19]. High EE, characterized by critical comments, hostility, or emotional over-involvement from a relative, can exert stress that negatively affects the overall functioning among individuals with mental disorders, leading to an increased relapse risk and compromised clinical recovery [20, 21].

While previous studies have shown that high EE is an established predictor of relapse or hospitalization among patients with schizophrenia and various psychiatric illnesses [22, 23, 24], the association of EE with relapse in BD remains uncertain, with the existing evidence being fragmented. An investigation into this association is essential, as it assists the development of a targeted, family-based intervention, such as family psychoeducation, which can reduce relapse rates and support long-term recovery through tackling the root of the suffering [25, 26]. Moreover, the exploration of this area is particularly important, given the substantial personal, social, economic, and healthcare burdens associated with individuals and the care of individuals with BD and relapse [27, 28, 29, 30]. Therefore, to bridge this gap, this review aims to systematically evaluate the available evidence in this area and identify any patterns of association between EE and relapse of BD. By clarifying the role of EE, this review seeks to contribute to more evidence-based and informed care strategies to improve outcomes and overall quality of life of individuals living with BD.

## 2. Methods

### 2.1 Eligibility Criteria

To maximize the number of studies included, any observational or clinical studies reporting the relationship between EE and relapse of BD, for example, randomized controlled trials, quasi-randomized trials, cross-sectional, case-control, retrospective, and prospective cohort studies. Studies were included if they met the following criteria: (1) Adult individuals with BD diagnosed using valid and modern diagnostic methods (e.g., Diagnostic and Statistical Manual of Mental Disorders, Fourth Edition or Fifth Edition (DSM-IV or DSM-V), International Classification of Diseases, Tenth Revision or Eleventh Revision (ICD-10 or ICD-11) or equivalent version of the international diagnostic manual), (2) outcome measures included the level of EE and bipolar relapse (or relapse-related outcome). The exclusion criteria were: (1) individuals diagnosed with BD have co-morbidity of long-term physical disabilities or neurodevelopmental conditions, (2) articles investigating BD with other psychiatric illnesses without reporting separate outcome data.

### 2.2 Information Sources

A comprehensive search was conducted in the following databases: MEDLINE (https://pubmed.ncbi.nlm.nih.gov/), Embase (https://www.embase.com), CINAHL (https://www.ebscohost.com/nursing/products/cinahl-databases/cinahl-complete), Web of Science (https://www.webofscience.com), PsycINFO (https://www.apa.org/pubs/databases/psycinfo), Scopus (https://www.scopus.com), and Cochrane Central Register of Controlled Trials (https://www.cochranelibrary.com/central). Additionally, two trial registries, ClinicalTrials.gov (https://clinicaltrials.gov/) and the ISRCTN Registry (https://www.isrctn.com/), were searched for ongoing studies. Secondary searches included Google Scholar (https://scholar.google.com/) and the reference lists of included articles. All sources were searched from their inception to 29 December 2025, and limited to English-language publications.

### 2.3 Search Strategy

The search strategies were developed using the Population, Exposure, Outcome (PEO) framework and Medical Subject Headings (MeSH) terms, in consultation with a university librarian. The primary search terms included “Bipolar” AND “Expressed emotion” AND “Relapse”, applied to title, abstract, and keyword fields. The search strategies for MEDLINE were provided as an example: (“BD”[MeSH] OR “Bipolar” OR “Manic-Depressive”) AND (“Expressed Emotion”[MeSH] OR “Family Conflict”) AND (“Recurrence”[MeSH] OR “Relapse” OR “Readmission”). Similar strategies were adapted for other databases, with filters for English-language articles and human studies. Secondary searches in Google Scholar used the same keywords, and reference lists of included studies were manually screened. The full search strategies across electronic databases and trial registries were attached in the **Supplementary Material**. This review was prospectively registered in PROSPERO (CRD42025632303) and adhered to the Preferred Reporting Items for Systematic Reviews and Meta-Analyses (PRISMA) 2020 checklist (**Supplementary Material**) [31].

### 2.4 Selection Process

Two independent reviewers (SYT, MCHD) screened titles and abstracts against the inclusion criteria. Potentially relevant studies were retrieved in full and assessed for eligibility. Discrepancies were resolved by a third reviewer (AHTY). The selection process was conducted independently, with no automation tools used. A PRISMA flow diagram was generated to document the number of records screened, assessed, and included.

### 2.5 Data Collection

Data were extracted by two independent reviewers (SYT, MCHD) using a standardized data extraction form. Extracted data included author, year of publication, study design, geographical location, sample size, participant characteristics (e.g., age, gender, bipolar subtype), assessment tools for EE, relapse measures, number of relapse events, time of follow-up, and methodological quality. Cross-checking was performed by the two reviewers (SYT, MCHD) to ensure the accuracy of the extracted data, and any discrepancies would be resolved by the third reviewer (AHTY).

### 2.6 Study Risk of Bias Assessment

Two independent reviewers (SYT, MCHD) critically appraised the eligible studies using the Joanna Briggs Institute (JBI) SUMARI version Sep 2022 (JBI, The University of Adelaide, Adelaide, SA, Australia) with adherence to the PRISMA guideline for systematic review, and the methodological quality of the included studies was assessed independently using standardized critical appraisal instruments from the JBI. As the included papers consisted of 6 non-experimental studies and 1 experimental study, different appraisal instruments corresponding to each study design were utilized.

For non-experimental studies, the JBI’s Critical Appraisal Checklist for Cohort Studies and the JBI’s Critical Appraisal Checklist for Analytical Cross-sectional Studies were used to assess the quality of cohort studies (*k* = 4) and a cross-sectional study (*k* = 2), respectively [32]. For experimental studies, the Revised JBI’s Critical Appraisal Tool for the Assessment of Risk of Bias for Randomized Controlled Trials was applied to appraise the included randomized controlled trial (*k* = 1) [33].

Each JBI’s critical appraisal checklist consists of 8 to 13 questions, depending on the study design. Each question was rated by two independent reviewers (SYT, MCHD) and could be scored as Met (yes), Unmet (no), Unclear, or Not Applicable. The included studies were operationally categorised into high, moderate, and low risk of bias (RoB), based on the number of ‘yes’ responses to the checklist questions. Low RoB would be given to studies with scores of ≥9 on the JBI’s checklist for cohort studies; ≥8 on the JBI’s Critical Appraisal Checklist for Cross-sectional Studies; ≥10 on the Revised JBI’s Critical Appraisal Tool for the Assessment of Risk of Bias for Randomized Controlled Trials. Moderate RoB would be given to studies with scores of 6 to 8 on the JBI’s checklist for cohort studies; 6 to 7 on the JBI’s Critical Appraisal Checklist for Cross-sectional Studies; 7 to 9 on the Revised JBI’s Critical Appraisal Tool for the Assessment of Risk of Bias for Randomized Controlled Trials. High RoB would be given to studies with scores below the thresholds of that from moderate RoB. These results were documented in the appraisal form, reported narratively, and summarised in a table. Any disagreements that arose during the quality review process were resolved by the third reviewer (AHTY).

### 2.7 Procedures for Data Synthesis

Results from the individual included studies were categorized into multiple sections to report the summary findings. A table was used to overview the included study characteristics and their key findings. The planned meta-analysis, subgroup analyses, and sensitivity analysis were not conducted due to the small sample size (i.e., ≤10 studies with retrievable data for pooling).

## 3. Results

### 3.1 Study Selection and Characteristics

#### 3.1.1 Overview of the Selected Studies

**Fig. 1.**
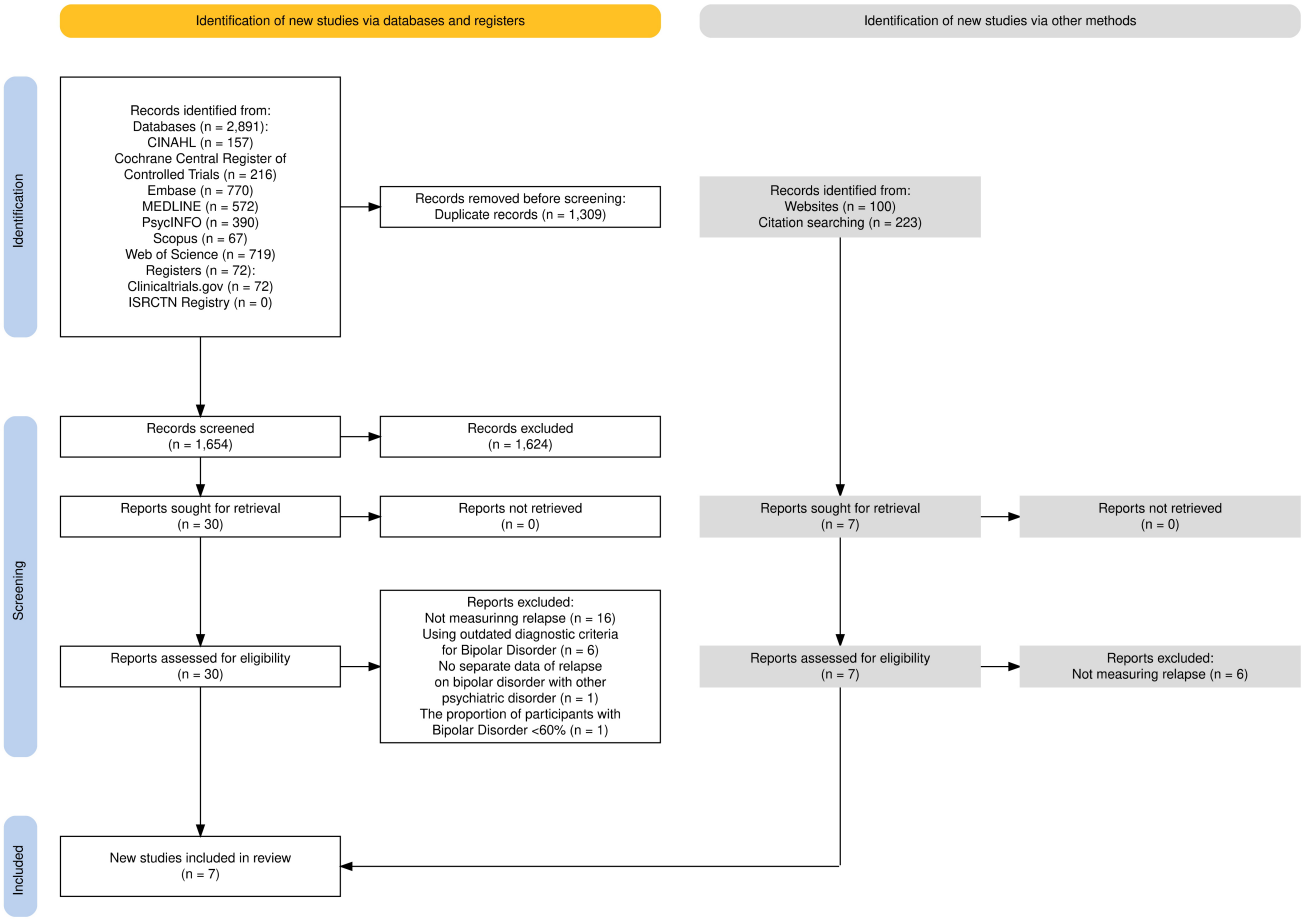
**PRISMA flow diagram**. PRISMA, Preferred Reporting Items for Systematic Reviews and Meta-Analyses.

The study selection process for this review is detailed in the PRISMA flow diagram (Fig. 1). A total of 2963 studies were identified from the database (*k* = 2891) and registries (*k* = 72) searches. After removing duplicate studies, 1654 studies were retained for title and abstract screening, and 30 studies were sought for retrieval. 30 studies entered full-text screening. 7 articles were eligible for inclusion in this review, while the remaining 23 articles were excluded for the following reasons: not measuring relapse (*k* = 16); using outdated diagnostic criteria for BD (*k* = 6); no separate data of relapse on BD with other psychiatric disorders; the proportion of participants with BD <60% (*k* = 1).

Additionally, 323 studies were identified via citation searches from the included articles and Google Scholar. Of the 7 eligible studies for full-text screening, 6 were excluded because they did not measure relapse events, while 1 was included. In total, 7 articles were included in this review, with 6 being non-experimental studies (i.e., 4 cohort studies; 2 cross-sectional studies) and 1 being an experimental study (i.e., Randomised controlled trial (RCT)).

#### 3.1.2 Study Characteristics

The year of publication of the included studies ranged from 2004 to 2025, with being conducted in the US (*k* = 2), Europe (*k* = 2) and Asia (*k* = 3). There were a total of 528 participants with BD were involved in the included studies: 321 were diagnosed with bipolar I disorder, 25 with bipolar II disorder, and for 182 participants, the type of BD was unspecified (Bipolar I: 60.8%, Bipolar II: 4.7%, unspecified: 34.5%). For the demographic data, there were more males (*n* = 276) than females (*n* = 252), with a weighted mean age of 40.9 years.

### 3.2 Measurement Tools Used for Expressed Emotion and Relapse

To assess the level of EE from relatives or friends, the Family Attitude Scale (FAS) (*k* = 3) was the predominant tool used. Other instruments included the Camberwell Family Interview (CFI) (*k* = 2), Perceived Criticism Scale (PCS) (*k* = 2), and Five-Minute Speech Sample (*k* = 1). Valid assessment tools, clinical evaluation, and hospital admission records were used to determine the occurrence of relapse episodes. Follow-up durations ranged from 9 months to 33 months, with 2 studies reporting 12 months, while the average attrition rate of the included prospective cohort studies and an RCT was 14.6% (standard deviation (SD) = 17.9%; range = 0–39.2%).

### 3.3 Narrative Summary Findings of the Included Studies

#### 3.3.1 Association of Expressed Emotion and Relapse or Symptom Exacerbation

Across the 7 available studies, a general trend suggested an association between high EE and increased relapse risk or symptom exacerbation in BD patients. For instance, high EE was significantly associated with depressive recurrence in BD (adjusted odds ratio (OR) = 5.39, *p* < 0.03), but not manic recurrence [34], while a similar association between high EE and higher levels of depressive symptoms was also noted (*p* = 0.03) [20]. Shimodera *et al*. [35] found a 100% relapse rate in the high EE group versus 0% in the Low EE group (*p* = 0.005). Both Endreddy *et al*. [36] and Verma *et al*. [37] conducted cross-sectional studies to reveal that there was a significant association between high EE and relapse (χ^2^ = 12.26, *p* < 0.01) or the number of hospitalisations (*r* = 0.433, *p* < 0.001).

#### 3.3.2 Subdomains of Expressed Emotion

The findings of the included studies showed that critical comments were more strongly associated with depressive relapses compared to manic relapses. A study conducted by Lex *et al*. [38] found that critical comments increased the risk of depressive recurrences in the supportive therapy group but not in the cognitive behavioural therapy group. Perceived critical comments, as measured by Scott *et al*. [39], were significantly correlated with hospital admissions (adjusted OR = 3.33, *p* = 0.004), especially when coupled with other risk factors, such as low medication adherence and poor family knowledge. However, emotional over-involvement showed mixed results, with Kim and Miklowitz [20] reporting no significant impact on relapse, while Shimodera *et al*. [35] linked emotional over-involvement ≥3 to relapse (*p* = 0.005).

#### 3.3.3 Source of Expressed Emotion

Four articles mentioned the categorization of EE, totalling 234 participants, in which 102 were categorized into the high EE group, and 132 were in the Low EE group [20, 34, 35, 36]. Another four articles described the EE experienced by the service users were mainly from spouses (45.6%) or parents (34.1%), totalling 340 relatives or friends [20, 34, 36, 37]. Within the high EE group, 67 of them were from the US [20, 34], while 35 of them were from Asia (India [36] and Japan [35]). High emotional over-involvement was significantly reported among parents than among the spouse (*p* = 0.004) [20], while high global EE was significantly reported among spouses than parents (64% vs 37.5%, *p* = 0.017) [36].

### 3.4 Quality Appraisal

For the quality appraisal, 5 studies were rated as high quality, and 2 were rated as moderate quality. The details of each included study and the results of the quality review are presented in Tables 1 (Ref. [20, 34, 35, 36, 37, 38, 39]), 2a (Ref. [20, 34, 35, 39]), 2b (Ref. [36, 37, 38]), respectively.

**Table 1.** **Summary of included studies on expressed emotion and relapse in BD**.

Study (Author, Year)	Design	Location	Baseline sample size	Mean age (years)	BD type	EE tool	Follow-up (months)	Relapse measure	Key findings
Kim & Miklowitz (2004) [20]	Prospective Cohort	USA	125	35.89	Bipolar I & II	CFI	24	SADS-C	High EE was not linked to faster relapse, but associated with higher depression severity (*p* = 0.03).
Yan *et al*. (2004) [34]	Prospective Cohort	USA	64	42.0	Bipolar I	FMSS	12	SCID	High EE predicted depressive recurrence (OR = 5.39, *p * < 0.03), not manic.
Scott *et al*. (2012) [39]	Prospective Cohort	UK	81	42.2	Bipolar I & II	PCS	12	Hospital admissions	High perceived criticism was correlated with hospital admissions (*p* = 0.004).
Shimodera *et al*. (2012) [35]	Prospective Cohort	Japan	12	49.7	Bipolar I	CFI	9	BPRS & HDRS	The high EE group had a 100% relapse rate vs 0% in the Low EE group (*p* = 0.005).
Lex *et al*. (2022) [38]	RCT	Germany	76	43.96	Bipolar I & II	FAS & PCS	33	Clinical interviews, diaries	Perceived critical familial attitudes were linked to depressive recurrence (HR = 1.03, *p* = 0.01).
Endreddy *et al*. (2024) [36]	Cross-sectional	India	50	43.8	Bipolar Affective Disorder	FAS	N/A	Prior relapse events	Significant association between high EE and relapse (χ^2^ = 12.26, *p * < 0.01).
Verma *et al*. (2025) [37]	Cross-sectional	India	120	85% of participants ranging from 21–40*	Bipolar Affective Disorder	FAS	N/A	Number of hospitalizations	High EE was significantly associated with the number of hospitalizations (*r* = 0.433, *p * < 0.001).

Footnote: BD, bipolar disorder; EE, expressed emotion; CFI, Ccamberwell family interview; AS, affective style; PSP, patient symptom profile; FMSS, five minute speech sample; PCS, perceived criticism scale; FAS, family attitude scale; BPRS, brief psychiatric rating scale; SADS-C, the schedule for affective disorders and schizophrenia-change version; SCID, structured clinical interview for DSM disorders; HDRS, hamilton depression rating scale; RCT, randomised controlled trial; OR, odds ratio; HR, hazards ratio. *The mean age was not provided in the article.

**Table 2a.** **Risk of bias of the included cohort studies**.

Study (Author, Year)	Design	Question number from the JBI’s Critical Appraisal Checklist											Score	Risk of bias
		1	2	3	4	5	6	7	8	9	10	11		
Kim & Miklowitz (2004) [20]	Prospective cohort	N	Y	Y	Y	Y	Y	Y	Y	Y	N	Y	9/11	Low
Yan *et al*. (2004) [34]	Prospective cohort	Y	Y	Y	Y	N	N	Y	Y	Y	NA	Y	8/11	Moderate
Scott *et al*. (2012) [39]	Prospective cohort	N	Y	Y	Y	Y	Y	Y	Y	Unclear	Unclear	Y	8/11	Moderate
Shimodera *et al*. (2012) [35]	Prospective cohort	Y	Y	Y	Y	N	Y	Y	Y	Y	NA	Y	9/11	Low

JBI, Joanna Briggs Institute; N, no; Y, yes; NA, not applicable.

**Table 2b.** **Risk of bias of the included non-cohort studies**.

Study (Author, Year)	Design	Question number from the JBI’s Critical Appraisal Checklist													Score	Risk of bias
		1	2	3	4	5	6	7	8	9	10	11	12	13		
Lex *et al*. (2022) [38]	RCT	Y	Y	Y	N	N	Y	Y	Y	Y	Y	Y	Y	Y	11/13	Low
Endreddy *et al*. (2024) [36]	Cross-sectional	Y	Y	Y	Y	Y	Y	Y	Y	-	-	-	-	-	8/8	Low
Verma *et al*. (2025) [37]	Cross-sectional	Y	Y	Y	Y	Unclear	Y	Y	Y	-	-	-	-	-	7/8	Low

## 4. Discussion

This study has examined the existing findings from primary studies to provide a comprehensive overview of the association between EE and relapse of BD. From our results, several key findings have been found. First, high EE appears to be significantly related to relapse, regardless of the relapse duration and stage of the disease. Similar results have also been reported in other psychiatric disorders such as schizophrenia, first episode psychosis, and depression. A meta-analysis conducted by Mazza and his colleagues [40] reported an overall OR of 2.09, suggesting that high EE may be a strong predictor of relapse in schizophrenia and major depressive disorder. Another review investigating the association between EE and the first episode of psychosis also found that high EE is significantly associated with relapse in first episode psychosis [41].

Beyond the finding that high EE may be associated with relapse among BD, our results revealed that, out of the five subdomains of EE, emotional over-involvement and critical comments are the two subdomains that are most potent in driving relapse. This finding is consistent with Keegan *et al*. [41], where emotional over-involvement and critical comments are the two most significant components correlated with relapse. Additionally, familial critical comments were directly correlated with relapse, while high perceived criticism by service users was also significantly linked to more frequent hospital admissions. This suggests that the patient’s perception of their family’s comments is as clinically relevant as the objectively measured critical comments themselves. The possible mechanism behind the stronger association between emotional over-involvement and critical comments with relapse is that these subdomains are more likely to intensify the negative perceptions in individuals with BD during emotional processing [42, 43]. Such intensified responses may increase the chance of emotional distress or exhaustion, making the individuals more prone to relapse [44].

Although the planned meta-analysis and subgroup analyses could not be performed due to insufficient data, the narrative synthesis of the included studies suggests a plausible pattern that high EE may demonstrate a stronger association with depressive episodes rather than manic episodes in BD. In a prospective cohort of 64 outpatients with Bipolar I disorder, the findings revealed that high EE significantly predicted depressive recurrence, but not manic recurrence [34]. Similarly, Kim and Miklowitz [20] have demonstrated that a higher level of critical comments predicted a higher level of depressive symptoms of service users across two-year follow-ups. Lex *et al*. [38] also echoed these findings, showing that relatives’ criticism ratings specifically predicted recurrences of depressive episodes, while no significant risks for manic episodes were observed. Moreover, the influence of EE extends beyond the risk of relapse to encompass the overall burden of illness. It tends to be linked to a significantly higher depression severity. However, the moderating effects of age, clinical stages, duration of condition, and EE tools used on the association between EE and relapse remain unclear.

Given that depressive episodes dominate the course of BD and the potential linkage between high EE and depressive relapse, the non-pharmacological treatment alleviating familial high EE should be a central target mechanism of change for the prevention of depressive relapse in BD [45]. Previous studies have demonstrated that medications alone cannot maximize the treatment effect on preventing recurrence of bipolar episodes, and psychotherapy can be implemented alongside to elicit protective effects against relapse [25, 46, 47]. Psychotherapies such as family-focused therapy have been used as an adjuvant treatment with medications and show positive results in minimizing relapse [7, 8, 26, 47, 48]. Given that EE may be a predictor of bipolar relapse, our findings provide both research and clinical practice directions for the development of family-focused therapy to lower the relapse rate of BD. Strategies can be established by modifying the components of EE among family members of bipolar patients. Negative attributions described by Hooley and colleagues [49] could be a potential target mechanism of change of family-focused therapy for high EE families, as caregivers may attribute patients’ negative behaviours, for example, social withdrawal and drowsiness, to internal and personal factors, rather than illness or adverse effects of medications. Family-focused therapy focusing on emotional regulation in high EE families may also be beneficial to reducing relapse by modulating caregivers’ reactions towards BD patients [50, 51]. Fredman *et al*. [52] have proposed the mechanism of family-focused therapy, where caregivers are educated in identifying symptom recurrence in BD patients, and in limiting self-sacrificing and over-involvement behaviours in families. Consequently, emotional distress displayed towards BD patients can be minimized to prevent relapse episodes. The findings from this review align with the proposed mechanism and the potential targets of family-focused therapy in existing literature, which reinforces the efficacy of family-focused therapy’s role in modifying EE to minimize BD relapse risks. The majority of studies included in this review were from Western societies; future studies may consider investigating family dynamics in other cultures or regions and their corresponding influence on EE and relapse of BD. Furthermore, future studies could provide a novel perspective by studying individual subdomains of EE in-depth or exploring the mechanism of how different subdomains of EE lead to relapses of BD, thereby providing a more comprehensive explanation at different analytic levels.

From our included studies, various instruments with different cutoffs and operational definitions were applied to determine the level of EE and relapse of BD, respectively. This measurement heterogeneity may limit the comparability across studies in detailed resolution. For example, Camberwell Family Interview assesses the EE in five subdomains: critical comments, hostility, emotional over-involvement, and two protective subdomains (i.e., warmth and positive comments), in which classification of EE is mostly based on any one of the first three subdomains [53], whereas Family Attitude Scale and Five Minute Speech Sample did not measure hostility or emotional over-involvement [54, 55]. Their global construct measurement was fundamentally similar: the attitudes of relatives or close friends towards individuals with psychiatric disorders. Although the Camberwell Family Interview is regarded as the gold standard for measuring EE, other EE measures are also valid and comparable in terms of concurrent validity and predictive validity [56]. Similarly, the operational definitions of relapse varied across the included studies across the globe. Some adopted clinical interviews or assessor-rated scales to measure relapse by cut-offs or changes in symptom exacerbation or deterioration [20, 34, 35, 38], while some adopted the number of psychiatric hospitalisations or readmissions based on medical records [36, 37, 39]. These differences were usually rooted in the study design, analysis or aim of the individual studies, depending on research resources and labour intensity. The International Society for Bipolar Disorder [57] reviewed that the use of clinical interviews (e.g., SCID), global rating scales (e.g., morbidity index, including hospitalization), and severity rating scales (e.g., Hamilton Rating Scale for Depression) are common in defining relapse, and they recommended to specify the period (i.e., >8 weeks after remission) to complement the definition of relapse, regardless of the index episode. In our included four cohort studies and 1 RCT, they followed up the participants for at least 9 months to suffice time for relapse. As various measures are used for relapse, there seems to lack a global consensus on such operational definitions, unlike a recent consensus definition of relapse in schizophrenia, including valid scales used (e.g., Positive and Negative Syndrome Scale), severity of symptoms (e.g., 12 points change on global score or 4 points change on subdomain scores), and duration (e.g., ≥1 week or require immediate intervention) [58]. Additionally, this recent systematic review and international consensus study also found that 81% of trials used hospitalization alone as an indicator of relapse [58], which may be a reliable proxy variable for immediate intervention to severely deteriorating symptoms.

For the mechanism of how EE relates to psychiatric relapse, the underlying biological processes are not yet fully understood. One possible explanation is the use of the consensus model of BD [59]. However, it should be viewed as theoretical, hypothesis-generating, rather than the conclusive findings generated from this review, as this model was based on multiple observational neuroimaging studies beyond the included studies. Drawing on the consensus model of BD and indirect evidence from neuroimaging studies, dysfunction in prefrontal-limbic circuits has been proposed as a potential pathway [59]. In healthy individuals, the prefrontal cortex modulates amygdala activity within the limbic system, contributing to emotional processing [59]. However, among individuals with BD, disruptions in white matter connectivity and pruning of the prefrontal area have been noted. These disruptions weaken the connection between the prefrontal-limbic network to regulate emotions [59, 60], and several functional Magnetic Resonance Imaging (fMRI) studies have reported greater amygdala activation in BD patients in response to negative emotional stimuli [61]. On this basis, when individuals with BD are exposed to high EE environments, they may perceive these situations as negative emotional tasks, stimulate the limbic system and cause over-activation of the amygdala [62]. Notably, the studies included in this review did not directly assess neural function, so this prefrontal-limbic account remains speculative and requires direct examination in future mechanistic research.

### Strengths and Limitations

Our review has several strengths. To ensure the completeness of our search, a wide variety of search terms were used by exploring MeSH terms and reviewing the keywords in relevant published articles related to our topic. Additionally, authors of the included studies were contacted to request more information on articles that were without full-text or raw data. Another strength was that to achieve a thorough understanding of the association between EE and bipolar relapse, the findings from different subdomains and sources of EE were synthesized and evaluated.

However, caution is required when interpreting the findings with the following limitations. Significant clinical and methodological heterogeneity within the included studies exists as the major limitation. Between-study heterogeneity manifests, for example, from different EE assessment tools, differing study designs, and relapse measures. The narrative synthesis from multiple studies using various EE instruments may incur incomparable findings at the levels of subdomains of EE. Diverse definitions of relapse employed by included studies also contribute to heterogeneity within our results. Also, our findings were limited by the information reported in the included studies. As the methods of presenting results varied among the included studies and some data were unretrievable, we were unable to pool the effect estimates from all included studies (*k* = 7). Similarly, the predominance of Western studies in this review limits the generalizability of our findings in other regions. López *et al*. [63] examined the cultural variability and its influence on EE among schizophrenia and depression, specifically in Anglo-American families and Mexican American families. Their findings suggested that culture affects the expression of emotions within families and family attitudes towards ill relatives. These results call for adapting EE measurements to the culture’s characteristics, like the substantial cultural differences between Mexican American, Asian and Jewish populations [63, 64]. Future studies may consider investigating family dynamics in other cultures or regions and their corresponding impact on EE and relapse of BD. Furthermore, the relapse frequency and time to relapse were underreported in the included studies, leading to limited resolution to understand this association. Additionally, the definitions of high EE and relapse were not unified across studies, which may contribute to the variation of findings. Given the observational nature of our analysis, the factors linking EE and BD relapse remain ambiguous. EE may also reflect illness severity, chronicity and caregiver burden, suggesting a potentially bidirectional or confounded relationship with various factors acting as a moderator, mediator, or confounder. Therefore, it is important to consider multiple factors when estimating BD relapse. Moreover, subdomains of EE were not investigated in most of the articles, making it difficult to interpret and conclude the findings regarding the relationship between different subdomains of EE and relapse in BD. Besides, as the number of prospective cohort studies on this topic was scarce, relevant cross-sectional and experimental studies were also included in the review to supplement a broader picture. The inclusion of treatment-related studies may interfere with our results by introducing other confounding factors. Finally, two studies with moderate RoB [34, 39] were included in the narrative synthesis. Respective RoB in the individual study should be carefully considered when interpreting the individual study’s findings.

## 5. Conclusions

This review underscores the potential association between high EE and the risk of relapse in BD by providing updated evidence. These findings are beneficial to fostering the development of care strategies aimed at reducing the level of EE from relatives and friends. Future primary studies are required to validate the association and to explore how different subdomains of EE impact the risk of relapse in BD.

## Availability of Data and Materials

All data used in this study are available from the corresponding published articles and their additional files.
