# Supplementary material for: Association Between Expressed Emotion and Relapse of Bipolar Disorder: A Systematic Review
Source: Alpha Psychiatry. 2026 Jun 2;27(3):47961. doi: 10.31083/AP47961 (PMC13339780; doi:10.31083/AP47961)
Supplement: Supplementary file 1 [file 2757-8038-27-3-47961-s1.zip › Supplementary Material.docx]

***Search Strategies in Each Electronic Database:***

**CINAHL:**

Results: 157

S1 (bipolar disorder): TI OR AB OR MH

S2 (expressed emotion): TI OR AB OR MH

S3 (relapse): TI OR AB OR MH

S4: combine S1 AND S2 AND S3

*Limited to English

TI ("Manic depress*" or Bipolar or "Affective psycho*") OR AB ("Manic depress*" or Bipolar or "Affective psycho*") OR MH bipolar disorder AND TI ("Expressed emotion*" or EE or Famil* or "Critical comments" or Hostility or "Positive remarks" or Warmth or Emotional over-involvement or "Camberwell Family Interview" or "Five-minutes speech sample" or EOI or CFI or FMSS or LEE or FQ) OR AB ("Expressed emotion*" or EE or Famil* or "Critical comments" or Hostility or "Positive remarks" or Warmth or Emotional over-involvement or "Camberwell Family Interview" or "Five-minutes speech sample" or EOI or CFI or FMSS or LEE or FQ) OR MH expressed emotion AND TI (recurrence or relapse or recrudescence or rehospitalization or readmission or exacerbation) OR AB (recurrence or relapse or recrudescence or rehospitalization or readmission or exacerbation) OR MH patient readmission OR MH recurrence

*Limited to English

**Cochrane Central Register of Controlled Trials:**

Results: 216

ti, ab, kw; MeSH descriptor, explode all trees

*Limited to English

((Manic NEXT depress* OR Bipolar OR Affective NEXT psycho*):ti,ab,kw OR

MeSH descriptor: [Bipolar Disorder] explode all trees)) AND (("Camberwell Family Interview" or "Five-minutes speech sample" or EOI or CFI or FMSS or LEE or FQ):ti,ab,kwOR MeSH descriptor: [Expressed Emotion] explode all trees) AND ((recurrence or relapse or recrudescence or rehospitalization or readmission or exacerbation):ti,ab,kw OR MeSH descriptor: [Recurrence] explode all trees)

**Embase:**

Results: 770

PICO search, ti, ab, kw, exp/mj

*Limited to English

('bipolar disorder'/exp/mj OR 'bipolar affective disorder':ti,ab,kw OR 'bipolar and related disorders':ti,ab,kw OR 'bipolar disorder':ti,ab,kw OR 'bipolar illness':ti,ab,kw OR 'bipolar psychosis':ti,ab,kw OR 'depression, manic':ti,ab,kw OR 'manic depression':ti,ab,kw OR 'manic depression psychosis':ti,ab,kw OR 'manic depressive':ti,ab,kw OR 'manic depressive disease':ti,ab,kw OR 'manic depressive disorder':ti,ab,kw OR 'manic depressive illness':ti,ab,kw OR 'manic depressive psychosis':ti,ab,kw OR 'manic depressive reaction':ti,ab,kw OR 'manic depressive syndrome':ti,ab,kw OR 'maniodepressive psychosis':ti,ab,kw OR 'mano depressive syndrome':ti,ab,kw OR 'psychosis, manic depressive':ti,ab,kw) AND ('emotion'/exp/mj OR 'expressed emotion':ti,ab,kw OR 'critical comment*':ti,ab OR 'hostility'/exp/mj OR 'hostility':ti,ab,kw OR 'positive remark*':ti,ab OR 'warmth':ti,ab OR 'emotional overinvolvement':ti,ab OR 'camberwell family interview':ti,ab OR 'five minute speech sample'/exp/mj OR 'eoi':ti,ab OR 'cfi':ti,ab OR 'fmss':ti,ab OR 'lee':ti,ab OR 'ee':ti,ab OR 'fq':ti,ab OR 'famil*':ti,ab) AND ('recurrent disease'/exp/mj OR 'recurrence':ti,ab,kw OR 'relapse'/exp/mj OR 'relapse':ti,ab,kw OR 'recrudescence'/exp/mj OR 'recrudescence':ti,ab,kw OR 'hospital readmission'/exp/mj OR 'hospital readmission':ti,ab,kw OR 'patient readmission':ti,ab,kw OR 'readmission':ti,ab,kw OR 'readmissions':ti,ab,kw OR 'rehospitalization':ti,ab,kw OR 'exacerbation'/exp/mj) AND [english]/lim

**MEDLINE:**

Results: 572

S1 (bipolar disorder): TI OR AB OR MH

S2 (expressed emotion): TI OR AB OR MH

S3 (relapse): TI OR AB OR MH

S4: combine S1 AND S2 AND S3

*Limited to English

TI ("Manic depress*" or Bipolar or "Affective psycho*") OR AB ("Manic depress*" or Bipolar or "Affective psycho*") OR MH bipolar disorder AND TI ("Expressed emotion*" or EE or Famil* or "Critical comments" or Hostility or "Positive remarks" or Warmth or Emotional over-involvement or "Camberwell Family Interview" or "Five-minutes speech sample" or EOI or CFI or FMSS or LEE or FQ) OR AB ("Expressed emotion*" or EE or Famil* or "Critical comments" or Hostility or "Positive remarks" or Warmth or Emotional over-involvement or "Camberwell Family Interview" or "Five-minutes speech sample" or EOI or CFI or FMSS or LEE or FQ) OR MH expressed emotion AND TI (recurrence or relapse or recrudescence or rehospitalization or readmission or exacerbation) OR AB (recurrence or relapse or recrudescence or rehospitalization or readmission or exacerbation) OR MH patient readmission OR MH recurrence

*Limited to English

**PsycINFO:**

Results: 390

S1 (bipolar disorder): TITILE OR AB OR IF (keyword)

S2 (expressed emotion): TITILE OR AB OR IF (keyword)

S3 (relapse): TITILE OR AB OR IF (keyword)

Combine S1 AND S2 AND S3

*Limited to English

("Manic depress*" or Bipolar or "Affective psycho*") AND ("Expressed emotion*" or EE or Famil* or "Critical comment*" or Hostility or "Positive remark*" or Warmth or Emotional over-involvement or "Camberwell Family Interview" or "Five-minutes speech sample" or EOI or CFI or FMSS or LEE or FQ) AND (recurrence or relapse or recrudescence or rehospitalization or readmission or exacerbation)

**Scopus:**

Results: 67

S1: TI AB KW

S2: combine with AND

*Limited to English

( ( TITLE-ABS-KEY ( "Expressed emotion*" OR ee ) OR TITLE-ABS-KEY ( famil* OR "Critical comment*" OR hostility OR "Positive remark*" OR warmth OR emotional AND over-involvement ) OR TITLE-ABS-KEY ( "Camberwell Family Interview" OR "Five-minutes speech sample" OR eoi OR cfi OR fmss OR lee OR fq ) ) ) AND ( TITLE-ABS-KEY ( "Manic depress*" OR bipolar OR "Affective psycho*" ) ) AND ( TITLE-ABS-KEY ( recurrence OR relapse OR recrudescence OR rehospitalization OR readmission OR exacerbation ) ) AND ( LIMIT-TO ( LANGUAGE , "English" ) )

**Web of Science:**

Results: 719

S1: Topic (TI AB KW)

*Limited to English

((TS=("Manic depress*" or Bipolar or "Affective psycho*" )) AND TS=("Expressed emotion*" or EE or Famil* or "Critical comment*" or Hostility or "Positive remark*" or Warmth or Emotional over-involvement or "Camberwell Family Interview" or "Five-minutes speech sample" or EOI or CFI or FMSS or LEE or FQ)) AND TS=(recurrence or relapse or recrudescence or rehospitalization or readmission or exacerbation)

***Search Strategies in Each Trial Registries:***

**Clinicaltrials.gov**

Results: 72

S1:

Condition/ Disease: bipolar disorder

Other Terms: “expressed emotion”

**ISRCTN Registry**

Results: 0

S1:

Condition/ Disease: bipolar disorder

Other Terms: “expressed emotion”
